# Nature‐based interventions to promote health for people with stress‐related illness: An integrative review

**DOI:** 10.1111/scs.13089

**Published:** 2022-05-23

**Authors:** Gunilla Johansson, Päivi Juuso, Åsa Engström

**Affiliations:** ^1^ Division of Nursing and Medical Technology, Department of Health, Education and Technology Luleå University of Technology Luleå Sweden

**Keywords:** health promotion, literature review, natural environment, nature‐based intervention, stress‐related illness

## Abstract

**Background:**

Stress‐related illness is increasing and is a common cause of sick leave. Spending time in nature have a positive effect on health and well‐being for instance by reducing stress. Specific programmes with nature‐based interventions (NBI) with the intention to involve people in activities in a supportive natural environment have been developed for people with stress‐related illness.

**Aim:**

To identify and summarise scientific studies of NBIs to promote health for people with stress‐related illness.

**Method:**

The design used in this study is integrative literature review. Scientific studies focusing on any type of NBI for people with stress‐related illness were sought in Cinahl, PubMed, PsycInfo, AMED and Scopus. In total, 25 studies using both qualitative and quantitative designs were included in the review.

**Result:**

The reviewed studies focused on garden or forest interventions. In the majority of the studies, NBIs were performed in groups, including individual activities, and the length of programmes varied. Interventions in natural environments have unique qualities for individualised, meaningful activities and interactions with others in a non‐demanding atmosphere. NBIs offer restoration that reduces stress, improves health and well‐being and strengthen self‐efficacy and work ability. Connectedness with nature support existential reflections and people with stress‐related illness can achieve balance in everyday life.

**Conclusion:**

In conclusion, NBIs may have advantages to promote health for people with stress‐related illness and should therefore be considered as an alternative to those affected. Further research from different perspectives, including nursing, is needed to understand the possibilities of NBIs and how they can be integrated into practice.

## INTRODUCTION

Contact with nature affects people's health and well‐being in a positive way, for example, by reducing stress [1]. Accordingly, specific programmes with nature‐based interventions (NBI) have been developed for people with stress‐related illness and studied by interdisciplinary research teams [2, 3, 4]. However, research from a nursing perspective about NBI for people with stress‐related illness is scarce. Only one of the 25 articles included in this review has one author within nursing. In this paper, we present an overview of scientific studies of NBI to promote health for people with stress‐related illness and discuss this in relation to among other aspects also to nursing.

## BACKGROUND

According to the World Health Organisation [5], health emphasises social and personal resources in people's everyday lives. McCartney et al. [6] define health as something experienced by individuals and a capacity to live as an individual and participate in society. These descriptions correspond with the definition of health used within nursing.

Mental illness is a main contributor to ill health globally and a considerable cause for people living with disability [7]. In industrialised countries, it is a main cause of sick leave [8, 9]. Mental illnesses include different diagnoses, such as stress‐related mental illness, which is increasing in Sweden, especially among women [10, 11, 12]. In this review, we focus on stress‐related illness due to people being exposed to stress for long periods without restoration, resulting in a lack of resources [13]. The most prevalent causes for stress‐related illness are high workload or emotional demands at work, but stressors in private life can also be a contributing factor [14]. Factors in the work environment such as low support and low job control increase the risk of stress‐related illness [15].

The core signs of stress‐related illness are tiredness and decreased energy levels. Physical symptoms such as gastrointestinal problems, headache, dizziness, chest pain and other bodily pain are also common [16], as well as sleep disturbances and cognitive difficulties such as impaired memory, concentration and executive function [17]. People with stress‐related illness can manage to perform despite cognitive difficulties but with a great effort, resulting in increased mental tiredness [18]. Stress‐related illness might have considerable long‐lasting consequences. People suffering from stress‐related illness can have cognitive difficulties, tiredness and problems related to daily activities many years after seeking care, even if they are considered to be recovered [19]. To achieve well‐being in everyday life when living with stress‐related illness, opportunities for unconditional beingness, that is not having demands on oneself and not having to perform, are required. Being in nature can provide such unconditional beingness and promote well‐being in everyday life [20].

Spending time in nature has been shown to have a positive effect on health and well‐being [1, 21], it enhances mood and attention capacity [22] and increases mindfulness and meaning [23]. Nevertheless, the importance of contact with nature is often overlooked in modern society [21].

Nature‐based interventions take their stance in this knowledge and aim to involve people in activities such as gardening, farming, physical activity or interaction with animals in a supportive natural environment [4]. The profession and role of staff working with NBI varies depending on the type of intervention. For example, staff can have qualifications within health care, psychotherapy, horticulture and/or agriculture [21]. Interprofessional teams are common but seldom include nurses [3, 4].

Several researchers have studied NBI and its effect on health. Kondo et al. [24] showed that spending time outdoors in green spaces reduces stress and improves health. In a systematic review, Annerstedt et al. [25] found that nature‐assisted therapy improved health for participants with various illnesses. Corazon et al. [26] showed that NBI decreased stress, anxiety and depression among the participants and improved well‐being and quality of life. Furthermore, Steigen et al. [27] found that NBI improved the participants' coping abilities, health, feeling of meaningfulness and dignity.

Stress‐related illness is complex, with psychological, physical and existential aspects, and people affected have described experiences of not being understood and respected by healthcare professionals. Such treatment can result in feelings of loss of control and lack of support, which can worsen the affected persons' condition [28, 29]. Thus, nursing with a holistic perspective and a focus on health has an important role in promoting health for people with stress‐related illness. In NBI, nurses' competence in caring relationships, supporting and empowering people to care for themselves might be a vital resource. If NBI is to complement traditional health care, the interventions and outcomes should be documented and compiled from a nursing perspective.

## AIM

To understand which NBI are used and their outcome for people with stress‐related illness, we aimed to identify and summarise scientific studies of NBI to promote health for people with stress‐related illness. The research questions were as follows: *Which NBI have been studied? What are the key elements in NBI for people with stress‐related illness? What are the experiences and outcomes of NBI?*

## METHOD

### Design

An integrative literature review was conducted to describe current knowledge about NBI for people with stress‐related illness. The framework for integrative reviews described by Whittemore and Knafl was followed as it allows a combination of diverse quantitative and qualitative data sources that contributes to varied perspectives of a phenomenon [30]. To improve the quality of the manuscript and ensure that all key areas of the study are presented, a reporting guideline, the PRISMA checklist, has been used [31].

### Search procedure

A literature search was conducted in Cinahl, PubMed, PsycInfo, AMED and Scopus databases in June 2020. Subject headings in the databases were identified and used in combination with own search terms. Each search term was first used separately and then combined with Boolean operators. Search terms and combinations are presented in Table 1. Limits in the literature search were English language and, when possible, academic journals. No time limit was used.

**TABLE 1:** Search terms used in the literature search

*Search terms covering stress‐related illness* combined with the Boolean operator OR	*Search terms covering NBI* combined with the Boolean operator OR
Stress, psychological (subject heading in Cinahl, PubMed, AMED)	‘nature based’
	‘green care’
Psychological Stress (subject heading in PsycInfo)	nature‐assisted
‘psychological stress’	Horticulture (subject heading in Cinahl, PubMed, AMED)
‘stress related’	
‘exhaustion disorder’	Horticultural Therapy (subject heading in PubMed, PsycInfo, AMED)
burnout	
‘burn out’	horticultur*
Burnout professional (subject heading in AMED)	Gardening (subject heading in AMED)
Occupational Stress (subject heading in PsycInfo)	garden*
‘occupational stress’	Animal Assisted Therapy (subject heading in PubMed, PsycInfo, AMED)
	‘animal assisted therapy’
	animal‐assisted
	‘forest based’
	‘care farm’
*Finally, the two combinations of search terms were combined with AND*	

**FIGURE 1:**
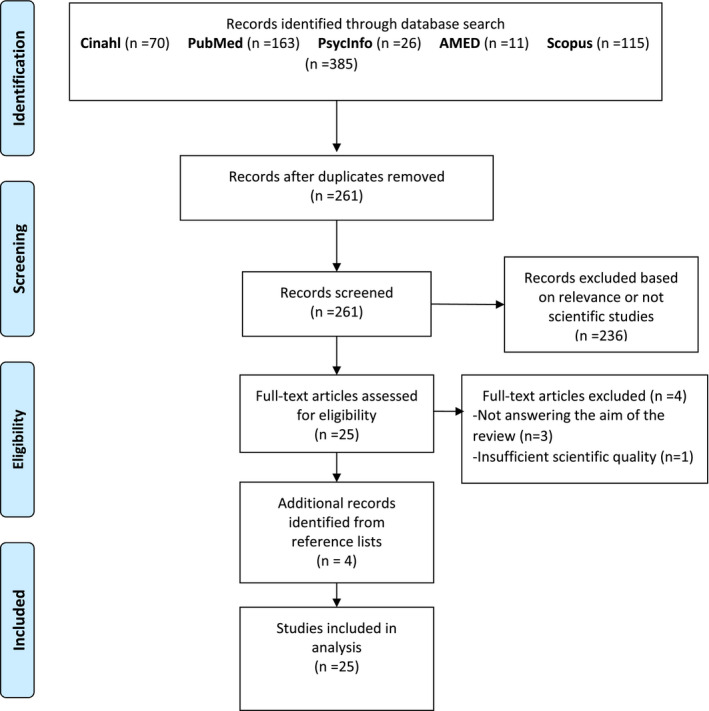
Flow diagram of the selection process

Inclusion criteria were original scientific studies written in English studying any type of NBI for people with stress‐related illness. Studies about people with post‐traumatic stress disorder were excluded. After literature searches, duplicates were excluded. The remaining records were screened by reading title and abstract to assess if they met the inclusion criteria. Matching articles were read in full text, and three articles were excluded based on not answering the aim of the review. The quality of each included study was assessed independently by two of the authors, using checklists from the Swedish Agency for Health Technology Assessment and Assessment of Social Services (https://www.sbu.se/en/method/) for different study methodologies. Disagreements were discussed among all authors until consensus was reached. One study was excluded due to insufficient scientific quality; there were few participants in the quantitative study. Reference lists of articles read in full text were reviewed, and four additional studies meeting the inclusion criteria were identified, quality assessed and included. In total, 25 studies published 2008–2018 were included in the analysis. The selection process is shown in Figure 1.

### Data analysis

Data from included studies were systematically extracted, coded, ordered, categorised and summarised [30]. All data answering the research questions were extracted and coded based on content. Coded data were organised in matrices, one for each research question, to facilitate the comparison of data from multiple primary sources. Data were categorised using an iteratively comparative approach to identify similarities and patterns in data. During the analysis, categories describing the types of interventions, key elements of NBI and experiences and outcomes were created. Included studies are described in Table 2 by author, year of publication, country, study design, participants, type of intervention and results. The result presentation is a summation in the form of categories answering the research questions.

**TABLE 2:** Description of studies included in the review, type of interventions and findings

Author (year) country	Study design	Participants (diagnosis/characteristics, Age, sex)	Type of intervention (control)	Results
Adevi & Lieberg (2012) [32] Sweden	Qualitative Semi‐structured individual interviews and focus‐group interview/ Grounded theory	5 members of a rehabilitation team	Garden therapy conversational therapy, physical therapy, relaxation, gardening and handicraft activities	Sensory impressions, self‐chosen places in the garden and interaction between symbolic and concrete activities were important for the stress recovery process
Adevi & Mårtensson (2013) [33] Sweden	Qualitative Semi‐structured interviews/ Grounded theory	Five participants with exhaustion disorder 25–60 years 4 women, 1 man	Garden therapy conversational therapy, physical therapy, relaxation, gardening and handicraft activities	Sensory experiences and symbolism of nature contributed to increased well‐being. The garden therapy offered opportunities that responded to participants' needs
Cerwén et al. (2016) [34] Sweden	Qualitative Semi‐structured interviews/ Interpretative phenomenological analysis (IPA)	59 participants with stress‐related mental disorders 25–62 years 50 women, 9 men	Nature‐based rehabilitation conversational therapy, physical therapy, relaxation, gardening and handicraft activities	Natural sounds were experienced as pleasant and supported recovery
Corazon et al. (2018) [35] Denmark	Quantitative Randomised controlled trial	84 participants with adjustment disorder and/or reaction to severe stress 43 nature‐based therapy (NBT) age m = 47.9 41 cognitive behavioural therapy (CBT) age m = 44.9 82% woman	Nature‐based therapy individual therapeutic conversations based on CBT, individual and group mindfulness exercise, individual and social garden activities, individual relaxation and reflection and homework	Both NBT and CBT led to decreased contacts with general practitioners and long‐term sick leave
			(CBT)	
Dolling et al. (2017) [36] Sweden	Quantitative Randomised controlled trial	46 participants with high stress levels 27 visited forest environment 19 visited handicraft environment age m = 48 33 women, 13 men	Forest environment relaxation around a fire, simple activities like walks or relaxation in solitude	Participants' health had improved in both forest and handicraft environments. Fatigue, stress and burnout were lower after intervention in both groups
			(Handicraft environment) relaxation, simple activities like wood carving, painting or relaxation in solitude	
Eriksson et al. (2010) [37] Sweden	Qualitative Semi‐structured interviews/ Grounded theory	7 workers at a rehabilitation clinic age m = 44, 6 women, 1 man Eight former clients with stress‐related disorders age m = 41, 7 women, 1 man	Therapeutic garden cognitive approach, therapy garden for half of the participants	Clients experienced changes in self‐image, development of strategies for handling stressful situations and changing occupational repertoire in everyday life
Eriksson et al. (2011) [38] Sweden	Qualitative Repeated semi‐structured interviews/ Grounded theory	Five women with stress‐related ill health 36–52 years old	Therapeutic garden cognitive training, relaxation and garden activities	A secure environment facilitated enjoyable activities that inspired participants in their everyday lives, contributing to occupational balance
Grahn et al. (2017) [39] Sweden	Quantitative Quasi‐experimental study	106 participants with reaction to severe stress and/or depression 22–63 years, m = 45.7, 96 women, 10 men	Nature‐based rehabilitation gardening activities, individual physical therapy and psychotherapy	Participants with longer rehabilitation period rated higher occupational competence and were more likely returned to work
Millet (2008) [40] Sweden	Quantitative Before‐after measure	32 women with exhaustion syndrome 22–63 years, md = 46	Vocational rehabilitation in garden environment using horticulture	Participants experienced better health, reduction of stress, increased energy and sleep improvements. Level of salivary cortisol was reduced after rehabilitation
Nordh et al. (2009) [41] Sweden	Mixed method Before‐after measure Participant observation Repeated individual interviews	24 participants with exhaustion syndrome or depression 27–61 years, m = 45 57% women	Forest environment activities, teaching and recreation	Participants enjoyed the programme and experienced physical and mental improvements. Symptoms of illness and general functioning improved, but not burnout scores
Pálsdóttir, Grahn et al. (2014) [42] Sweden	Mixed method Before‐after measure Semi‐structured interviews/ Qualitative content analysis	21 participants with adjustment disorder and/or reaction to severe stress or depression 29–68 years, m = 47, 19 women, two men	Nature‐based vocational rehabilitation occupational therapy, physiotherapy, psychotherapy and horticultural therapy	Occupational values increased, symptoms of stress decreased, and many participants had returned to work. The occupational repertoires in everyday life changed
Pálsdóttir, Persson et al. (2014) [43] Sweden	Qualitative Semi‐structured interviews/ Interpretative phenomenological analysis (IPA)	43 participants with adjustment disorder and/or reaction to severe stress or depression 25–62 years, m = 45.5, 35 women, eight men	Nature‐based rehabilitation programme occupational therapy, physiotherapy, psychotherapy and horticultural therapy	Rehabilitation process included three phases: *Prelude, Recuperating* and *Empowerment* supported by natural environments, rehabilitation team and other participants
Pálsdóttir, et al. (2018) [44] Sweden	Qualitative Narrative interviews and location mapping/ Narrative description	59 participants with stress‐related mental illnesses, 50 women, nine men	Nature‐based rehabilitation programme occupational therapy, physiotherapy, psychotherapy and horticultural therapy	The rehabilitation garden was a restorative environment. Social quietness is an important quality of supportive environment
Sahlin et al. (2014) [45] Sweden	Mixed method Before‐after measure Semi‐structured interviews/ Qualitative content analysis	33 women with sign of stress 45% ≤49 years 55% ≥50 years	Nature‐based stress management course garden activities, guided walks, handicraft, relaxation, group conversation, information about stress and health	Burnout scores, long‐term sick leave and stress‐related symptoms decreased, and work ability increased. The garden and nature were important for stress relief and for developing tools and strategies
Sahlin et al. (2015) [46] Sweden	Quantitative Before‐after measure, Quasi‐experimental	Participants with stress‐related mental illness 57 nature‐based rehabilitation (NBR) 26–63 years, m = 45, 53 women, four men 45 occupational health service (OHS) 32–61 years, m = 49, all women	Nature‐based rehabilitation garden activities, guided walks, handicraft, relaxation, group conversation, information about stress and health	Burnout, depression and anxiety decreased, well‐being increased after nature‐based rehabilitation. Health care utilisation was reduced in both groups
			(Occupational health service ‐ physical activity, counselling, medication, sick leave, dialogue with employer)	
Sahlin et al. (2012) [47] Sweden	Qualitative Semi‐structured interviews/Interpretative phenomenological analysis (IPA)	11 participants with exhaustion disorder and/or depression and anxiety 26–61 years, m = 43 eight women, three men	Nature‐based therapy physiotherapy, occupational therapy, psychotherapy, garden activities	It was important to be in the right phase of recovery when starting nature‐based therapy. Experiencing existential dimensions gave new perspective on life and change of dysfunctional patterns made it easier to handle everyday life
Sidenius, Karlsson Nyed et al. (2017) [48] Denmark	Mixed method Before‐after measure Semi‐structured interviews, behaviour mapping, logbooks/Qualitative content analysis	14 participants with adjustment disorder or/and reaction to severe stress, 20–60 years	Nature‐based therapy individual conversation therapy based on CBT, garden activities, awareness exercise, own time and homework	The therapy garden was experienced as protective and safe, with meaningful spaces and activities. Health increased
Sidenius, Stigsdotter et al. (2017) [49] Denmark	Qualitative Repeated semi‐structured interviews/ Reflective lifeworld analysis	14 participants with adjustment disorder or/and reaction to severe stress, 20–60 years	Nature‐based therapy individual conversation therapy based on CBT, physical and mental awareness exercise, garden activities, own time and homework	When participants became familiar with the garden, they felt safe to try new activities. They developed coping strategies to implement in everyday life
Sonntag‐Öström, Nordin et al. (2015) [50] Sweden	Quantitative Randomised clinical trial	99 participants with exhaustion disorder 51 forest rehabilitation 48 control group (on waiting list for stress rehabilitation clinic) age m = 44.6 85 women, 14 men	Forest rehabilitation breakfast and relaxation around a fire, 2 h in solitude in a forest setting, lunch by the fire (Both groups received cognitive behavioural rehabilitation after the period with forest rehabilitation)	Forest rehabilitation did not enhance recovery from exhaustion disorder compared to the control group, but mental state improved after single forest visits
Sonntag‐Öström et al. (2011) [51] Sweden	Mixed method Before‐after measure, Interviews/ Qualitative content analysis	Six participants suffering from stress‐related exhaustion 41–57 years, m = 49 three women, three men	Forest environment breakfast and mindfulness exercise around a fire, 2 h in solitude in a chosen forest setting, lunch by the fire	Solitude and various forest settings to meet individual preferences are positive factors for recovery. Forest visits had positive effects on participants' mental state, but fatigue did not decrease
Sonntag‐Öström, Stenlund et al. (2015) [52] Sweden	Qualitative Semi‐structured interviews/ Grounded theory	19 participants with exhaustion disorder 16 women 29–60 years, m = 49 three men 34–58 years, m = 44	Forest‐based rehabilitation repeated forest visits	After frustration at first, participants found favourite places and peace of mind. That led to reflective thinking and ambitions to change their life situation
Stigsdotter et al. (2018) [53] Denmark	Quantitative Randomised controlled trial	84 participants with adjustment disorder or reaction to severe stress 43 nature‐based therapy (NBT) age m = 47.9, 31 women, 7 men 41 cognitive‐behavioural therapy (CBT) age m = 44.9, 27 women, 6 men	Nature‐based therapy individual therapeutic conversations based on CBT, individual and group awareness exercise, individual nature‐based activities, individual relaxation and reflection and homework	Both treatments resulted in increased well‐being and decrease in burnout that sustained 12 months later. There was no difference between NBT and CBT
			(CBT ‐indoors)	
Tenngart Ivarsson & Grahn (2010) [54] Sweden	Qualitative Semi‐structured interviews/ Thematic analysis	10 participants with stress‐related diseases	Nature‐assisted therapy	Two themes emerged: ‘to escape, observe and get sensory stimulation’ and ‘to achieve satisfaction, socialise and re‐evaluate’
Willert et al. (2014) [55] Denmark	Quantitative Comparative pre‐post intervention design	93 participants on sick leave due to stress 48 garden programme age 25–59, m = 45.3, 38 women, 10 men 45 stress & job management programme age 26–59, m = 44.7, 39 women, six men	Garden therapy educational and physical activities, mindfulness and yoga outdoors or in greenhouse.	There was improvement in sleep, mindfulness, self‐efficacy, daily functions and work ability in both groups, but no difference between groups
			(Stress & job management programme ‐same activities but indoors)	
Währborg et al. (2014) [56] Sweden	Quantitative, Retrospective cohort study with matched reference group	103 participants with reactions to severe stress and/or depression age m = 45.9, 89% women, 11% men 678 matched controls age m = 46.3, 88% women, 12% men	Nature assisted rehabilitation gardening activities, relaxation exercise, psychotherapeutic activities, walking, most of the time outdoors	There was a reduction in healthcare consumption among participants in nature‐assisted rehabilitation compared with control group, but no difference in sick‐leave status

### Ethics

In this study, there has been no direct involvement with participants. Ethical approval was not required.

## RESULTS

**FIGURE 2:**
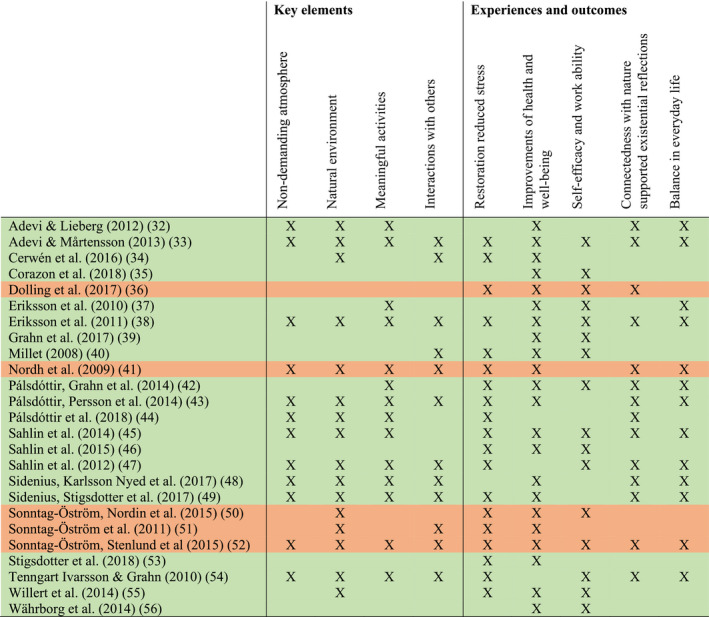
Categories with information of studies supporting each category. Garden interventions (green), Forest interventions (beige/brown)

The results are presented as a summation answering each research question based on included studies in accordance with Figure 2.

### Characteristics of included studies and types of interventions

Of the reviewed studies (*n* = 25), 17 are within agricultural science [32, 33, 34, 36, 39, 41, 42, 43, 44, 45, 46, 47, 50, 51, 52, 54, 56], and 10 are associated with health science [37, 38, 42, 43, 44, 47, 49, 50, 51, 52]. Eleven had a qualitative study design [32, 33, 34, 37, 38, 43, 44, 47, 49, 52, 54], nine had a quantitative design [35, 36, 39, 40, 46, 50, 53, 55, 56], and five had a mixed method design [41, 42, 45, 48, 51] (see Table 2). Different NBI programmes have been studied (see Table 2), and interventions differ regarding settings and activities. *Garden interventions* (*n* = 20, see Figure 2) [32, 33, 34, 35, 37, 38, 39, 40, 42, 43, 44, 45, 46, 47, 48, 49, 53, 54, 55, 56] took place in garden environments with gardening activities, and some also included nature‐like areas. *Forest interventions* (*n* = 5, see Figure 2) [36, 41, 50, 51, 52] took place in forest environments with calm activities. In the majority (*n* = 21) of the studies [32, 33, 34, 35, 36, 38, 39, 41, 42, 43, 44, 45, 46, 47, 48, 49, 50, 51, 53, 55, 56], NBI was performed in groups, including individual activities. In the remaining four studies [37, 40, 52, 54], the description of if NBI was performed individually or in groups was unclear. The duration of each occasion varied between 3 and 7 h, for two to 5 days a week, and the programmes lasted between six and 28 weeks.

### Key elements in NBI

**FIGURE 3:**
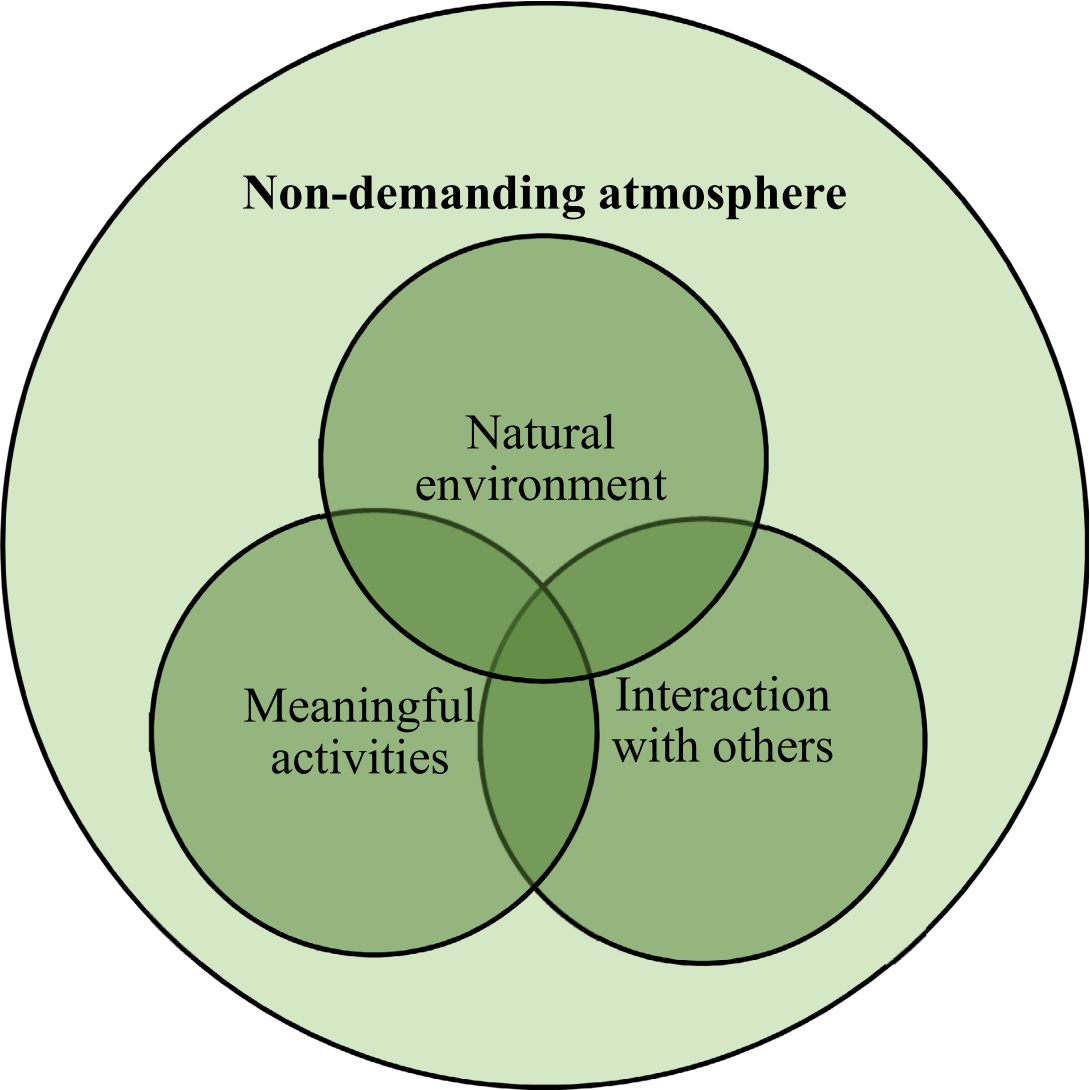
Categories of key elements and main category

The analysis revealed three categories of key elements within an overall main category (Figure 3). The results are presented as a summation of each category.

#### Non‐demanding atmosphere

In the reviewed studies, people with stress‐related illness described the place for NBI as a safe refuge [41, 44, 45, 54] separated from everyday life [38, 44, 49]. Participants could focus on the presence [43] with no external expectations [49] or demands [32, 33, 48, 49, 52, 54]. NBI was stimulating [33, 38, 48, 52], with various opportunities [33, 49] for participants to make own choices [54] in accordance with their personal needs and interests [33, 48, 49]. Nature offered a possibility to walk away and escape from demanding tasks and social interactions [33, 54]. The interplay between human, environment and activity supported the participants' process of recovery [48]. There was a permissive atmosphere [38, 41, 43, 44, 47], and guidance from staff [38, 48, 49] helped participants to use nature‐based components for personal development [49].

#### Natural environment

According to reviewed studies, the natural environment created a framework [32, 43, 47, 49] with variation and opportunities different from indoor settings [32, 33, 43, 54, 55]. It was calm [34, 38, 51, 52], peaceful [41, 44, 51] and offered possibilities for rest [43, 44]. Having the opportunity to choose places in nature based on emotional needs [32, 43, 44], mood [32, 33, 43, 52] and earlier experiences [48, 51, 52] was important for the participants. Proximity to water [50, 51, 52] and places with a good view [51, 52] was often preferred and secluded places where participants were hidden but could see others [43, 44, 48, 49, 52, 54]. Being outdoors in different weather conditions and following the seasons was appreciated [41], although some disliked rain and cold weather [48, 52]. Greenhouses could be an alternative offering shelter from bad weather [44, 54]. Senses were stimulated through sight of beautiful landscapes [33, 34, 41, 43, 44, 51, 52, 54], natural sounds such as running water and singing birds [34, 43, 44, 48, 51, 52, 54], smell of nature [44], taste of berries [44] and touch of nature [34, 43]. It was important that sensory stimuli were balanced to not be experienced as demanding [43, 44, 45, 54]. Noise from nearby traffic could be disturbing [34, 43, 54].

#### Meaningful activities

During NBI, participants could engage in meaningful activities that suited their current needs, moods and capabilities [47, 48, 49]. There were opportunities to be physically active, physically or mentally challenged [48, 49] or choose mentally restorative activities [48]. The activities could be gardening [33, 44, 54], cooking [33], walking [41], chopping wood [48], making fire [41, 52], handicrafts [47] or petting animals [32]. Participants did one thing at a time [42, 45, 47], without rushing [47] and without focusing on performance and results [32, 38, 43, 54]. There was, however, no pressure to take part in activities [48, 54] and they could enter and leave activities as they wanted [32, 43, 48]. The staff reminded the participants about taking breaks [45, 47] and minimising performance‐based behaviour [47]. Homework to integrate new strategies into everyday life justified spending time on activities for own recovery [37].

#### Interaction with others

In reviewed studies, participants described that they became part of a social context [33, 41, 47], were confirmed by staff [47] and got social support [40]. Meeting others in the same situation reduced feelings of being alone [33, 47] and gave opportunities to discuss everyday problems [41, 51]. They did not need to explain their condition, the others understand their difficulties [43]. The participants learned from others' experiences [47] and meeting others who was further in recovery gave hope [47, 54]. However, some were distressed by social situations such as gatherings with the group [34, 52]. Being able to choose when to interact with others or not was important [34] as social needs and capabilities varied overtime [49]. Time in solitude was valuable [38, 48, 51, 52, 54]; however, some were distressed about being alone in nature [52].

### Experiences and outcomes of nbi

The analysis resulted in five categories describing experiences and outcomes of NBI of people with stress‐related illness. The results are presented as a summation of each category based on included studies in accordance with Figure 2.

#### Restoration reduced stress

Reviewed studies showed that NBI offered stress relief [43, 45, 47], and participants learned to recognise signs of stress and how to use nature to cope with stress [45]. Participants could rest from worries in everyday life [47] and restore with energy [40, 43, 54]. Nature's rhythm and seasons gave perspective on time [33] and made them aware of the need for recovery [42, 47], influencing them to slow down the pace [34, 42, 43, 44, 47]. Taking part in NBI improved their relaxation [33, 36, 38, 43, 45, 47, 49, 50, 51, 52] and mindfulness skills [45, 55]. Participants expressed improvements in sleep [33, 52], which also was shown in quantitative studies [40]; however, most of them did not show statistically significant effect [36, 45, 55]. Some participants found it tiring to follow a schedule, go to the place and be part of a group [41]. Stress levels decreased after NBI [36, 40, 50, 55], which, however, also was shown in control groups [36, 50, 55]. One study [40] showed that salivary cortisol levels were reduced after NBI. Level of burnout decreased after NBI [36, 45, 46, 50, 53] which was also shown in some control groups [36, 50, 53]. One study [41] showed no statistically significant changes in burnout after NBI. Level of fatigue decreased after NBI both in intervention groups and control groups [36, 50]. In one study [51], no statistically significant difference was found in the level of fatigue.

#### Improvements of health and well‐being

Participants in reviewed studies reported positive effects on physical and mental health after NBI [36, 40, 41, 42, 48]. Sense of coherence [39, 42] and well‐being [33, 38, 45, 46, 48, 49, 52, 53] increased, although in one study, there were no difference from another stress treatment [53]. The general level of functioning increased, which also was shown after another stress treatment [55]. Fewer cognitive problems were reported [48] and fewer stress‐related physical symptoms, that is gastrointestinal symptoms, dizziness and heart palpitations [45]. Consumption of medicines [36, 40] and health care decreased, with fewer healthcare contacts [35, 46, 56] after NBI, which also was shown in one control group [35]. Mood improved [33, 41, 52], and feelings of pleasure, joy [32, 33, 38, 41, 43], satisfaction [33] and happiness [38, 41, 43, 52] were evoked. Positive memories were awakened [34, 38, 51], and participants felt more optimistic [37, 38, 43, 48], alert and harmonious [36, 50]. Reviewed studies showed decrease in depression and anxiety scores after NBI [46, 50]. In one study [50], no significant differences were found compared with the control group.

#### Self‐efficacy and work ability

In the reviewed qualitative studies, participants' expressed improvements in self‐esteem [33, 52], self‐efficacy [47], self‐image [37, 47] and self‐confidence [52] after NBI. One quantitative study found improvement in level of self‐esteem after NBI, but no difference was found compared with a control group [50] and another study found no significant improvement in self‐esteem [36]. Self‐efficacy was improved after NBI, but no difference was found compared with a control group [55]. The participants experienced restoration of self‐identity [47] and they realised their value was not dependent on performance [38, 47]. Feelings of competence [38, 54], self‐acceptance and motivation to change their situation [47] were evoked. Self‐assessed work ability [45, 55] and occupational competence [39] increased after NBI. In one study, work ability increased also after an indoor rehabilitation programme [55]. Sick leave decreased [35, 39, 40, 42, 46], and most of participants returned to work or job training [35, 39] after NBI. Three studies found no difference in sick leave compared with control groups [35, 50, 56]. Return to work was higher after longer nature‐based programmes [39]. Some participants attended job training in gardens or planned for education in horticulture [42].

#### Connectedness with nature supported existential reflections

Reviewed studies showed that participants attending NBI experienced meaningfulness [32, 33, 48] and a sense of belonging in nature [32, 43, 47, 48, 49], that they were part of something greater [32]. More time was spent outdoors in nature [42, 47, 52] with a newfound interest [38, 45, 47] and knowledge [45]. Participants found beauty in nature which they had not perceived before [33, 36, 45] and enjoyed and valued being in nature more than before NBI [33, 41, 45]. They experienced an underlying need for and connectedness with nature [43, 44, 47, 49, 52] and felt comforted in their vulnerable situation [43]. To be in nature gave peace in mind [44, 48] and opportunity for existential thoughts [32, 33, 45, 47] and personal reflections [44, 48, 54]. They could reflect on their life situation and found new perspectives [32, 47, 49, 52] and values in life [47]. Symbolism between processes in nature and their lives helped them understand their situation [47] and stimulated reflections of their need to take care of themselves and their personal growth [32, 33, 38, 43, 54].

#### Balance in everyday life

The reviewed studies found that participants developed individual tools and strategies to cope with challenges [33, 43, 45, 47, 48, 49] and take control of their everyday life [32, 37]. Participating in NBI helped them to establish daily routines [41, 49]. They started to establish new habits [48], although some struggled to implement new insights into everyday life [38, 52]. Participants were inspired to try new creative activities [38, 42, 43, 47, 49]. When they understood the importance of recovery, they achieved a better balance between activity and rest [37]. They introduced new undemanding and enjoyable activities [37, 38, 45] or took up old hobbies [42, 47, 54], but it could be hard to prioritise such newfound activities [38, 45].

## DISCUSSION

In this integrative review, we aimed to identify and summarise scientific knowledge about NBI to promote health for people with stress‐related illness. The reviewed studies had different research designs, with a focus on NBI programmes within garden or forest environments. We found that natural environments have unique qualities for individualised, meaningful activities and interactions in a non‐demanding atmosphere. NBI offered restoration that reduced stress, improved health and well‐being and strengthened self‐efficacy and work ability. Connectedness with nature supported existential reflections and people with stress‐related illness achieved balance in everyday life.

The natural environment is one of the main differences in NBI compared with other treatments of stress‐related illness. Our results showed that nature offers opportunities for individualised activities. According to McCormack et al. [57], a flexible environment makes it possible to individualise care, and opportunities to connect with nature and be outdoors are desirable. Anåker et al. [58] suggest that nature plays an important role in people's needs and desires and should be considered in health care environments to offer flexibility for patients' process to recovery.

The results in this review emphasise the possibility for people with stress‐related illness to choose settings, activities and interactions with others on their own terms to meet their current needs and capabilities. Supportive environment theory has been used to explain how people with stress‐related illness interact with outdoor environments in NBI. The theory combines environmental, social engagement and well‐being aspects. People with low well‐being tend to have a greater need for a supportive, safe and stable environment with limited stimulation, like nature. Due to their decreased capacity for social engagement, they may need the opportunity to be alone. Nature is described as an especially suitable environment for meaningful activities, offering challenges at different levels from passive experiences of nature to active engagement, for instance, gardening [3, 59].

The individualised approach of NBI can be related to person‐centred nursing. The goal of person‐centred nursing is satisfaction with care, involvement in care and feeling of well‐being. Professional competence such as knowledge and communication skills as well as the care environment are important, including the appropriate skill mix [60]. According to Ekman et al. [61], an important part of person‐centred nursing is to understand the persons' expectations, experiences, meanings and feelings to be able to establish a personal health plan based on the person's resources and capabilities [61]. Taken together, NBI and person‐centred nursing both focus on the person's individual needs and capabilities and promote health in partnership with the person. Nurses' knowledge, skills and experience with person‐centred nursing can be a valuable resource in NBI teams to provide holistic care.

The non‐demanding atmosphere of NBI shown in our results can be understood by Kaplan's [62] attention restoration theory. Directed attention used when concentration is needed requires energy and can, without restoration, result in attentional fatigue. Natural environments reduce the fatigue of directed attention by spontaneous attention. Soft fascination, an important component of spontaneous attention, occurs when something spontaneous catches and holds a person's interest without effort, like sensory impressions in natural settings [62]. This can explain the experiences of restfulness in natural environments found in the review.

In this review, we found that NBI offer restoration, decrease stress and promote well‐being for people with stress‐related illness. According to Anåker et al. [58], a supportive environment in health care reduces stress and promotes well‐being. An aesthetic environment with sensory and emotional stimulation promotes relaxation [57]. We found that nature helped persons with stress‐related illness slow down the pace. According to Dahlberg et al. [63], the distinct rhythm in nature with seasonal and diurnal variations can promote a healthy rhythm in life.

Our results showed that in addition to reduced stress, participants attending NBI felt connectedness with nature. Grahn et al. [64] emphasise people's experiences of calm and connection in nature. In nature, there is change and a movement showing life, and at the same time, there is stability and stillness. The hormone oxytocin released by positive experiences in nature reduces stress, increases well‐being and promotes affinity to nature. Being in nature can make people feel harmony, trust and a sense of belonging, making it possible to develop health and coping skills.

According to the results of this review, NBI enhances coping strategies and self‐identity for people with stress‐related illness. According to McCormack et al. [57], qualities of care environments are interconnected with a person's sense of self, sense of being and sense of connection with the world. Knowledge among healthcare personnel about the person's values and beliefs is important in person‐centred care. If the focus is on how illness impacts the person's life, the care can be individualised to meet his/her needs.

A recently published synthesis of qualitative studies shows that people with stress‐related illness participating in NBI experience positive effects on health and recovery. They described feelings of calm and joy, they said nature met their needs, and they found new insights and experienced personal growth [65]. Our results show that NBI supported existential reflections and personal development. According to Blekinsop [66], who refers to Buber, it is important to be aware of people's connection with nature. Nature is consistent and always available throughout our life, and deep encounters with nature can be important for personal development [66]. Based on this, we find that NBI can offer restoration and stress relief during the intervention and affect people's health and everyday life afterwards.

This review shows that people participating in NBI introduced restorative activities in their lives, which they continued after the programme. In a study [67], people with stress‐related illness reflected about moments of well‐being. Many reflections were about being outdoors in nature, which gave insights into the importance of spending time with such activities. Dahlberg et al. [64] describe health as an experience of balance in life, including movement and stillness. Movement means being able to do meaningful activities and realise projects in life. Stillness means rest and tranquillity but not necessarily inactivity. Stimulating activities without demands and focusing on performance can be a form of rest. Alternating between movement and stillness is sought, as disrupted balance can lead to ill health [64]. Stress‐related illness is an example of disrupted balance, with too many and too long periods of movement with a lack of stillness. According to our results, NBI can offer opportunities for restful activities and stillness without demands. Newfound interests and activities during NBI can be a resource to keep balance in everyday life.

According to Orem, nursing should create opportunities for people to promote their own health by strengthening their self‐care capacity. When an individual's needs are greater than their capacity, nursing should compensate for what the person cannot manage on their own and strengthen the person to regain the capacity to satisfy their own needs. To experience health, one universal self‐care need identified by Orem is to maintain a balance between rest and activity [68]. NBI can be a way to compensate for needs people with stress‐related illness cannot satisfy themselves. At the same time, they attain knowledge and strategies to find and maintain balance in life in a way that strengthens their self‐care ability and health.

Within nursing, it is known that contact with nature and viewing nature can promote health and well‐being. However, it is not often used in health care [69]. Some interventions, such as views of nature, natural sounds or natural elements to improve the environment and counteract stressors in hospital settings, have been tested [70]. Increased awareness of NBI for people with stress‐related illness and experiences and outcomes thereof can promote such interventions in nursing and be a complement to other treatments.

### Strengths and limitations

In this integrative review, a literature search was conducted in five databases, four associated with health and nursing. In the field of NBI, a lot of different terms are used. To capture relevant articles, literature within the field was searched prior to the literature search to find as many relevant search terms for NBI as possible. Thereafter, the different terms found were used in the systematic literature search. However, an extended search in more databases and with additional search terms could have resulted in finding an even larger number of relevant articles. There were no time restrictions in the literature search, which could lead to including old studies that do not reflect actual situations and knowledge. Even so, the oldest article was published in 2008, quite recently. All included studies are from two Nordic countries, Sweden and Denmark. A reason can be that the diagnosis of stress‐related health issues differs between different countries. The nature‐based programme in the studies is quite long‐lasting, and most participants were on long‐term sick leave. Health insurance policies in different countries may affect the possibilities to participate in NBI programmes. The regulation of using NBI for treatment also differs between countries. More than half of the included studies are published in same two journals which may be due to the field of research so far is relatively unexplored. Many of the quantitative studies included in this review did not show statistically significant effects of NBI which may be due to the low number of participants.

## CONCLUSION

Interventions in natural environments have unique qualities for individualised, meaningful activities and interactions with others in a non‐demanding atmosphere. NBI offer restoration that reduces stress, improves health and well‐being and strengthen self‐efficacy and work ability. Connectedness with nature support existential reflections and people with stress‐related illness can achieve balance in everyday life. Even though NBI are shown to promote health for people with stress‐related illness, further research is needed to better understand the possibilities, experiences and outcomes of NBI to suggest NBI as a nursing intervention.

## AUTHOR CONTRIBUTIONS

GJ, ÅE and PJ were involved in study design; GJ performed the literature search (with the help of a librarian at the university) and selection of articles; GJ, PJ and ÅE performed quality assessment of included articles; GJ, ÅE, PJ were involved in analysis and interpretation of data; GJ, ÅE and PJ were involved in the writing of the manuscript. All authors have agreed on the final version of this manuscript.

## CONFLICT OF INTEREST

The authors declare that there is no conflict of interest.

## ETHICAL APPROVAL

None.
